# Distinctive cell‐free DNA methylation characterizes presymptomatic genetic frontotemporal dementia

**DOI:** 10.1002/acn3.51997

**Published:** 2024-03-13

**Authors:** Lucia A. A. Giannini, Ruben G. Boers, Emma L. van der Ende, Jackie M. Poos, Lize C. Jiskoot, Joachim B. Boers, Wilfred F. J. van IJcken, Elise G. Dopper, Yolande A. L. Pijnenburg, Harro Seelaar, Lieke H. Meeter, Jeroen G. J. van Rooij, Wiep Scheper, Joost Gribnau, John C. van Swieten

**Affiliations:** ^1^ Department of Neurology, Alzheimer Center Erasmus MC Erasmus University Medical Center Rotterdam The Netherlands; ^2^ Department of Developmental Biology Erasmus Medical Center Rotterdam The Netherlands; ^3^ Department of Clinical Chemistry, Amsterdam Neuroscience Amsterdam UMC, Vrije Universiteit Amsterdam The Netherlands; ^4^ Erasmus Center for Biomics Erasmus University Medical Center Rotterdam The Netherlands; ^5^ Alzheimer Center Amsterdam, Neurology Vrije Universiteit, Amsterdam UMC location Vumc Amsterdam The Netherlands; ^6^ Amsterdam Neuroscience, Neurodegeneration Amsterdam The Netherlands; ^7^ Department of Functional Genomics, Center for Neurogenomics and Cognitive Research, Faculty of Science Vrije Universiteit Amsterdam The Netherlands; ^8^ Department of Human Genetics Vrije Universiteit, Amsterdam UMC location Vumc Amsterdam The Netherlands

## Abstract

**Objective:**

Methylation of plasma cell‐free DNA (cfDNA) has potential as a marker of brain damage in neurodegenerative diseases such as frontotemporal dementia (FTD). Here, we study methylation of cfDNA in presymptomatic and symptomatic carriers of genetic FTD pathogenic variants, next to healthy controls.

**Methods:**

cfDNA was isolated from cross‐sectional plasma of 10 presymptomatic carriers (4 *C9orf72*, 4 *GRN*, and 2 *MAPT*), 10 symptomatic carriers (4 *C9orf72*, 4 *GRN*, and 2 *MAPT*), and 9 healthy controls. Genome‐wide methylation of cfDNA was determined using a high‐resolution sequencing technique (MeD‐seq). Cumulative scores based on the identified differentially methylated regions (DMRs) were estimated for presymptomatic carriers (vs. controls and symptomatic carriers), and reevaluated in a validation cohort (8 presymptomatic: 3 *C9orf72*, 3 *GRN*, and 2 *MAPT*; 26 symptomatic: 7 *C9orf72*, 6 *GRN*, 12 *MAPT*, and 1 *TARDBP*; 13 noncarriers from genetic FTD families).

**Results:**

Presymptomatic carriers showed a distinctive methylation profile compared to healthy controls and symptomatic carriers. Cumulative DMR scores in presymptomatic carriers enabled to significantly differentiate presymptomatic carriers from healthy controls (*p* < 0.001) and symptomatic carriers (*p* < 0.001). In the validation cohort, these scores differentiated presymptomatic carriers from symptomatic carriers (*p* ≤ 0.007) only. Transcription‐start‐site methylation in presymptomatic carriers, generally associated with gene downregulation, was enriched for genes involved in ubiquitin‐dependent processes, while gene body methylation, generally associated with gene upregulation, was enriched for genes involved in neuronal cell processes.

**Interpretation:**

A distinctive methylation profile of cfDNA characterizes the presymptomatic stage of genetic FTD, and could reflect neuronal death in this stage.

## Introduction

Frontotemporal dementia (FTD) is a neurodegenerative disease comprising a set of clinical syndromes, with prominent behavioral and/or language deficits,^1^, ^2^ all characterized by frontotemporal neurodegeneration.^3^, ^4^ These syndromes result from the neuropathological aggregation of proteins in the brain, such as the tau and TDP‐43 proteins, accounting for approximately 40% and 50% of all FTD cases, respectively.^5^ A subset of FTD has a genetic etiology, due to autosomal dominant gene defects in the *MAPT* gene, associated with tau pathology, or in the *C9orf72*, *GRN*, or *TARDBP* genes, all associated with TDP‐43 pathology.^6^

Fluid biomarkers play a crucial role in the early diagnosis and prognostic follow‐up of patients with FTD syndromes,^7^ to improve clinical care and the design of clinical trials targeting the early disease stage.^8^, ^9^ Currently, neurofilament light chain (NfL), as the only established disease‐onset biomarker, is used to predict the onset of symptomatic disease.^10^ However, NfL is a nonspecific marker of neurodegeneration,^11^ and thus provides limited insight into specific biological processes implicated in FTD, or its genetic and pathological subtypes. Novel biomarkers with cellular or biological specificity are needed that reflect the underlying processes and help to track the complex disease dynamics of FTD.^7^

Cell‐free DNA (cfDNA) is freely circulating DNA shed from dying cells into the peripheral circulation.^12^, ^13^ Study of cfDNA has already been applied in other fields for the detection of fetal DNA abnormalities, as noninvasive prenatal testing,^14^ and for the diagnosis of cancer through liquid biopsies.^15^, ^16^ Through the identification of (epi‐)genetic alterations specific to the tissue of origin, cfDNA is a promising tool for the early detection and clinical follow‐up of many diseases,^17^, ^18^ including neurological diseases.^19^, ^20^ Recently, increased levels of plasma cfDNA have been found in Alzheimer's disease^21^ (AD), suggesting that cfDNA could be used as a peripheral marker for neurodegeneration, too.

DNA methylation is an epigenetic modification regulating gene expression, cell functioning and tissue differentiation, which has been associated with the development of different diseases,^22^ including neurodegenerative disorders.^23^, ^24^, ^25^ Differential methylation of brain tissue, peripheral blood mononuclear cells or non‐specific blood DNA has been identified in AD^26^, ^27^, ^28^ and in other types of dementia,^29^, ^30^ including FTD.^31^, ^32^, ^33^, ^34^, ^35^, ^36^, ^37^, ^38^, ^39^, ^40^ Methylation analysis can also be applied to cfDNA, to understand its tissue origin,^17^, ^18^, ^19^ as shown extensively in the oncology field.^41^ This feature has the potential to connect cfDNA to the underlying biological processes involved in the disease, such as neurodegenerative mechanisms in FTD.^19^ While prior studies found specific methylation signatures of cfDNA associated with AD,^21^, ^42^ methylation of cfDNA has never been studied in FTD.

In this novel explorative study of cfDNA methylation in genetic FTD, we aimed to investigate genome‐wide methylation of cfDNA in presymptomatic carriers of genetic FTD pathogenic variants, symptomatic carriers, and healthy controls. We used an innovative methodology, MeD‐seq, which enables to interrogate >50% of the entire DNA methylome,^43^ to uncover sites of differential methylation in an unbiased manner. Based on prior findings in dementia and other neurological disorders,^20^, ^21^ we expected carriers of genetic FTD pathogenic variants to show an enrichment of neuronal cells in plasma cfDNA, as opposed to the nonneuronal profile of cfDNA in healthy controls (primarily blood cells, erythrocyte progenitors, and endothelial cells).^44^

## Methods

### Subjects

We obtained cross‐sectional samples from participants of our longitudinal at‐risk cohort for genetic FTD, which follows at‐risk individuals from families with genetic FTD (FTD‐RisC cohort),^45^ and of patients with genetic FTD clinically diagnosed and treated at the Erasmus University Medical Center. Symptomatic carriers were identified as those (1) having cognitive symptoms/signs on clinical history or clinical evaluation (global CDR® plus NACC FTLD^46^ [FTLD‐CDR] ≥ 0.5), (2) showing an impairment (i.e., ≥1.5 SD below age‐, sex‐, and education‐specific means) or a substantial decline relative to a prior measurement in at least one domain on neuropsychological assessment, and (3) fulfilling criteria for FTD‐spectrum diagnoses of primary progressive aphasia (PPA),^2^ behavioral variant frontotemporal dementia (bvFTD),^1^ or FTD in combination with amyotrophic lateral sclerosis (FTD‐ALS).^47^ The study was approved by the Medical Ethics Review Committee of the Erasmus University Medical Center, and the study procedures were performed in accordance with the local ethical regulations. All participants provided informed consent to participate in the study.

First, we obtained a derivation cohort, including 10 presymptomatic carriers (4 *C9orf72*, 4 *GRN*, and 2 *MAPT*) and 10 symptomatic carriers (4 *C9orf72*, 4 *GRN*, and 2 *MAPT*). As cfDNA methylation may vary throughout disease stages, clinical groups of the derivation cohort were selected based on strict clinical criteria to investigate well‐defined presymptomatic/symptomatic stages: FTLD‐CDR = 0 and no clinical suspicion of incipient disease for presymptomatic carriers; 1–5 years of disease duration and FTLD‐CDR ≥1 for symptomatic carriers. Further, groups were matched for age (range 40–69 years) and for the distribution of variant‐carrying FTD genes. Additionally, we included an independent cohort of 9 healthy controls (5 females and 4 males) obtained via the Dutch National Blood Bank (Sanquin), already published elsewhere.^48^ All individuals within the derivation cohort were unrelated (Table S1).

Next, we reevaluated the methylation findings in a validation cohort including 8 presymptomatic carriers (3 *C9orf72*, 3 *GRN*, and 2 *MAPT*), 26 symptomatic carriers (7 *C9orf72*, 6 *GRN*, 12 *MAPT*, and 1 *TARDBP*), and 13 noncarriers of the FTD‐RisC study (i.e., first‐degree relatives of genetic FTD patients not carrying the disease‐causing variant). For these subgroups of the validation cohort, no strict selection criteria were applied, besides the basic criteria to define symptomatic carriers as opposed to presymptomatic carriers, as described above (i.e., FTLD‐CDR ≥0.5, decline on neuropsychological examination and conformity with FTD‐spectrum clinical criteria). A subset of 8 individuals within the validation cohort was related to each other (as first‐degree family members), and 9 individuals of the validation cohort were related to individuals of the derivation cohort (Table S2).

### Blood processing and cfDNA isolation

For participants of the FTD‐RisC cohort, blood was collected using either EDTA or CellSave (Menarini Silicon Biosystems, Castel Maggiore, Italy) blood tubes, which are both effective for cfDNA isolation and suitable for MeD‐seq analysis.^48^ Due to higher efficacy of isolations performed with CellSave tubes, we chose to use these tubes for newly collected samples (16 presymptomatic, 11 symptomatic, and 11 noncarriers, collected from August 2020 onwards), while previously collected samples from our biobank had been collected using EDTA tubes. Plasma was isolated from collection tubes within 24 h (EDTA) or 96 h (CellSave) after collection using a two‐step centrifugation procedure at room temperature (10 min at 1600 g followed by 10 min at 11,500 g at 4°C). Samples were subsequently stored at −80°C. For healthy controls of the Dutch National Blood Bank, blood was collected using CellSave tubes, and underwent similar processing procedures (two‐step centrifugation: 10 min at 1711 g followed by 10 min at 12,000 g). cfDNA was isolated from 2 to 3 mL of plasma using the manual QIAamp circulating nucleic acid kit (Qiagen), and eluted in the buffer AVE (RNase‐free water with 0.04% NaN_3_) provided by the kit. A quantity of 10 ng of isolated cfDNA was deemed sufficient to proceed to MeD‐seq analysis.

### MeD‐seq assay

MeD‐seq assays were essentially performed as previously described.^43^ In short, 8 μL genomic DNA plasma‐derived cfDNA samples were digested with LpnPI (New England Biolabs, Ipswich, MA) yielding 32 bp fragments around the fully methylated recognition site containing a CpG. Samples were prepped for sequencing using the ThruPLEX DNA‐Seq 96D kit (Rubicon Genomics, Takara Bio Europe, Saint‐Germain‐en‐Laye, France) and purified on a Pippin HT system with 3% agarose gel cassettes (Sage Science, Beverly, MA). Libraries were multiplexed and sequenced on an Illumina HiSeq 2500 for 50 bp single reads according to the manufacturer's instructions (Illumina, San Diego, CA).

### Data analysis

MeD‐seq data were processed and analyzed with Python 3.9.11 using customized scripts as described previously.^43^ Briefly, the raw FASTQ files were subjected to Illumina adaptor trimming and filtered for the presence of LpnPI restriction sites 13–17 bp from the 3′ or 5′ end. Next, reads were mapped to the Hg38 genome using bowtie 2.1.0, BAM files were generated using SAMtools and visualized using IGV 2.11.2. LpnPI site scores were used to produce read count scores for the transcription start sites (TSS; 1 kb before and 1 kb after), gene bodies (1 kb after the TSS until the transcription end site), and CpG islands, based on reference annotations from the UCSC (hg38).

To detect differentially methylated regions (DMRs), a genome‐wide sliding window was used to detect sequentially differentially methylated LpnPI sites between two groups (presymptomatic vs. healthy controls; presymptomatic vs. symptomatic; symptomatic vs. healthy controls) of the derivation cohort, genome‐wide read counts were normalized (RPM, reads per million) for coverage and compared using the chi‐squared test, with significance set at *p* < 0.05 and a Bonferroni correction for multiple testing. Neighboring significantly called LpnPI sites were binned and reported. DMRs located on the X‐ and Y‐chromosome were removed to avoid sex‐related effects. Overlap of genome‐wide detected DMRs was reported for TSS, CpG island or gene body regions using the annotations of UCSC (Hg38). DMR thresholds were based on LpnPI site count, DMR sizes (in bp), and fold changes of read counts before performing clustering.

In order to determine the presence or absence of group‐associated cfDNA methylation signatures per sample, receiver operating characteristic (ROC) curves were calculated for each individual DMR. For this analysis, we focused on the group of presymptomatic carriers (vs. healthy controls and vs. symptomatic carriers), as these showed the most prominent differential methylation. ROC curves of reference sets of presymptomatic derivation samples, compared to either control or symptomatic derivation samples, were used to calculate the optimal threshold (using the “scikit‐learn” package Python) for each individual DMR. Samples above the threshold scored 1, samples under the threshold scored 0. A cumulative score was generated for all DMRs resulting in hypermethylation scores associated with each group. Cumulative DMR scores were then compared between the groups in both derivation and validation cohorts using the Mann–Whitney *U*‐test.

### Protein–protein interaction analysis

Protein–protein interaction analysis was performed to identify the biological relevance of involved methylation sites (including all TSS and gene bodies overlapping with the identified DMRs) using the STRING database (https://string‐db.org), v. 11.5.^49^ Methylated sites in TSS and gene bodies were tested separately, as TSS methylation is generally associated with reduced gene expression, while gene body methylation is generally associated with increased gene expression.^50^ For each comparison (presymptomatic vs. controls; presymptomatic vs. symptomatic), genes with a fold change ≥2 were included. Only canonical gene transcripts were used for the analysis. In the STRING search tool, PPI networks were constructed using the “Homo sapiens:9606” species as reference, an interaction score >0.4 (i.e., medium confidence), and the following interaction sources: experiments, databases, co‐expression, gene fusion, and co‐occurrence (excluding text mining and neighborhood as interaction sources). Significant terms for biological processes, molecular functions and cellular components, based on the Gene Ontology database, were identified. The PPI network was visualized using STRING built‐in network viewer.

## Results

### Cohort characterization

The derivation cohort included 10 presymptomatic carriers (4 *C9orf72*, 4 *GRN*, and 2 *MAPT*), 10 symptomatic carriers (4 *C9orf72*, 4 *GRN*, and 2 *MAPT*), and 9 healthy controls with similar baseline demographic features (Table 1). The symptomatic carriers had clinical diagnoses of bvFTD (8/10, 80%), FTD‐ALS (1/10, 10%) or PPA (1/10, 10%), median disease duration of 2.1 years and median FTLD‐CDR score of 1.5. In the validation cohort, the 8 presymptomatic carriers (3 *C9orf72*, 3 *GRN*, and 2 *MAPT*), 26 symptomatic carriers (7 *C9orf72*, 6 *GRN*, 12 *MAPT*, and 1 *TARDBP*), and 13 noncarriers had similar baseline demographic features, too (Table 1). Clinical diagnoses of the symptomatic carriers were bvFTD (23/26, 88.5%), FTD‐ALS (1/26, 3.8%), and PPA (2/26, 7.7%); median disease duration was 2.4 years and median FTLD‐CDR score was 2.

**Table 1:** Baseline features of the derivation and validation cohorts.

Derivation cohort	Presymptomatic carriers	Symptomatic carriers	Healthy controls	Sig.
	*n* = 10	*n* = 10	*n* = 9	
Age	50.6 (48.1–59.6)	54.6 (52.2–61.5)	28.0 (25.0–52.0)	0.170
Female	6/10 (60%)	6/10 (60%)	5/9 (55.6%)	0.975
Variant‐carrying gene				
*C9orf72*	4/10 (40%)	4/10 (40%)		
*GRN*	4/10 (40%)	4/10 (40%)		
*MAPT*	2/10 (20%)	2/10 (20%)		
FTLD‐CDR	0 (0–0)	1.5 (1.0–2.0)	n/a	0.000
Age at onset		53 (48–57)		
Disease duration		2.1 (1.3–3.4)		
Clinical diagnosis				
bvFTD		8/10 (80%)		
FTD‐ALS		1/10 (10%)		
PPA		1/10 (10%)		

Validation cohort	Presymptomatic carriers	Symptomatic carriers	Non‐carriers	Sig.
	*n* = 8	*n* = 26	*n* = 13	
Age	59.8 (46.6–64.5)	58.2 (53.6–66.7)	57.2 (48.7–58.8)	0.522
Female	5/8 (62.5%)	12/26 (46.2%)	8/13 (61.5%)	0.560
Variant‐carrying gene				
*C9orf72*	3/8 (37.5%)	7/26 (26.9%)		
*GRN*	3/8 (37.5%)	6/26 (23.1%)		
*MAPT*	2/8 (25%)	12/26 (46.2%)		
*TARDBP*	0/8 (0%)	1/26 (3.8%)		
FTLD‐CDR	0 (0–0.5)	2.0 (1.0–2.0)	0 (0–0)	0.000
Age at onset		55 (49–59)		
Disease duration		2.4 (1.3–6.6)		
Clinical diagnosis				
bvFTD		23/26 (88.5%)		
FTD‐ALS		1/26 (3.8)		
PPA		2/26 (7.7%)		

Continuous variables are reported with median and interquartile range, and compared between groups using the Kruskal–Wallis test (presymptomatic vs. symptomatic vs. healthy controls/noncarriers). Categorical variables are reported as N/total and percentages, and compared between groups using the chi‐squared test.

bvFTD, behavioral variant frontotemporal dementia; FTD‐ALS, frontotemporal dementia in combination with amyotrophic lateral sclerosis; FTLD‐CDR, global clinical dementia rating (CDR)® plus National Alzheimer's Coordinating Center (NACC) frontotemporal lobar degeneration (FTLD); n/a, not available; PPA, primary progressive aphasia; Sig., significance.

### Distinctive methylation profile in presymptomatic carriers

**Figure 1:**
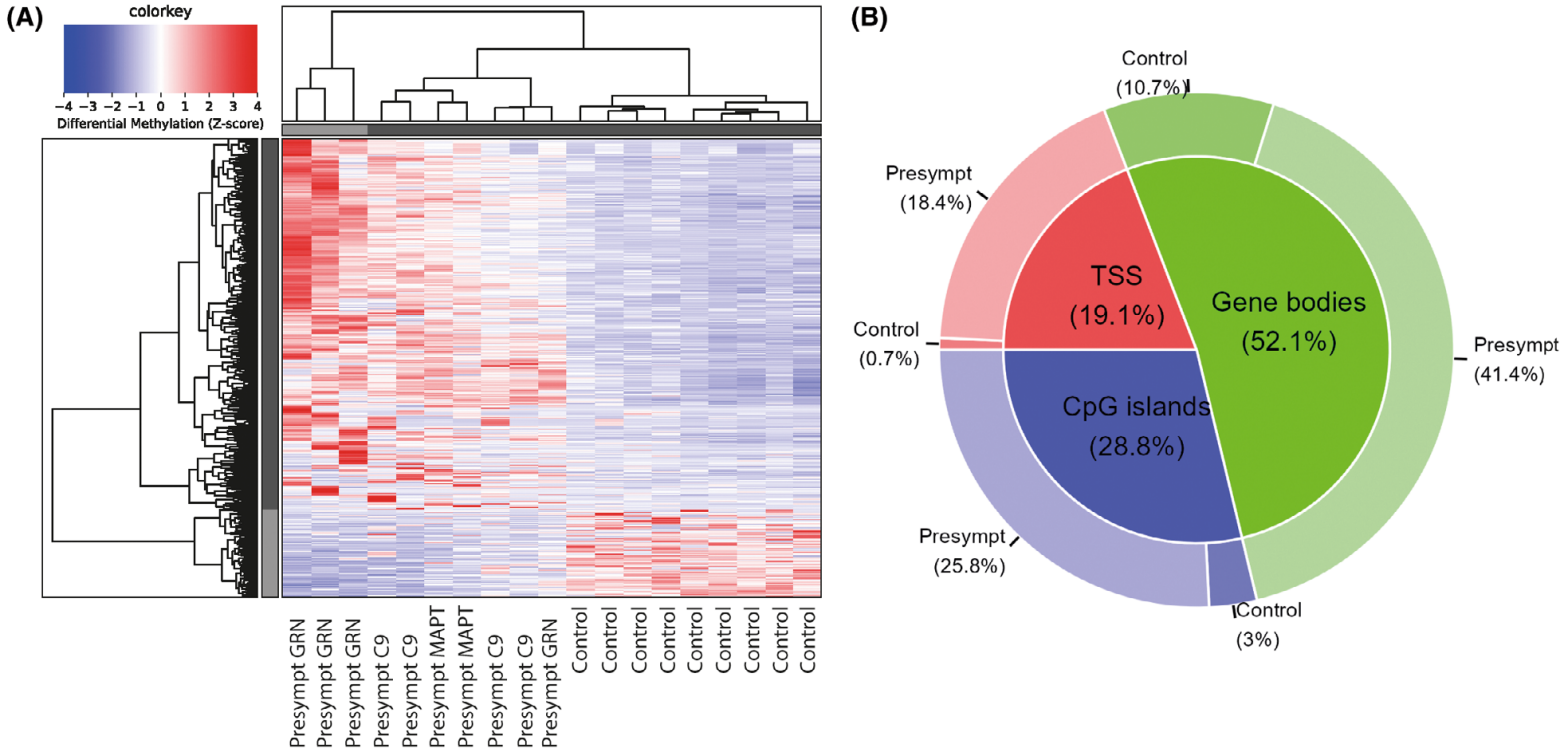
Differentially methylated regions in presymptomatic carriers versus healthy controls. In the comparison of presymptomatic carriers versus healthy controls, 838 significant differentially methylated regions (DMR) with a fold change ≥2 were identified, of which 678 hypermethylated in presymptomatic carriers and 160 in healthy controls (A). These DMRs included TSS (19.1%), gene bodies (52.1%) and CpG islands (28.8%) (B). Clustering shown in figure was performed based on a differential methylation z‐score, including DMRs with a fold change ≥2, excluding DMRs located on the X and Y chromosomes. C9, *C9orf72* gene; Presympt, presymptomatic.

**Figure 2:**
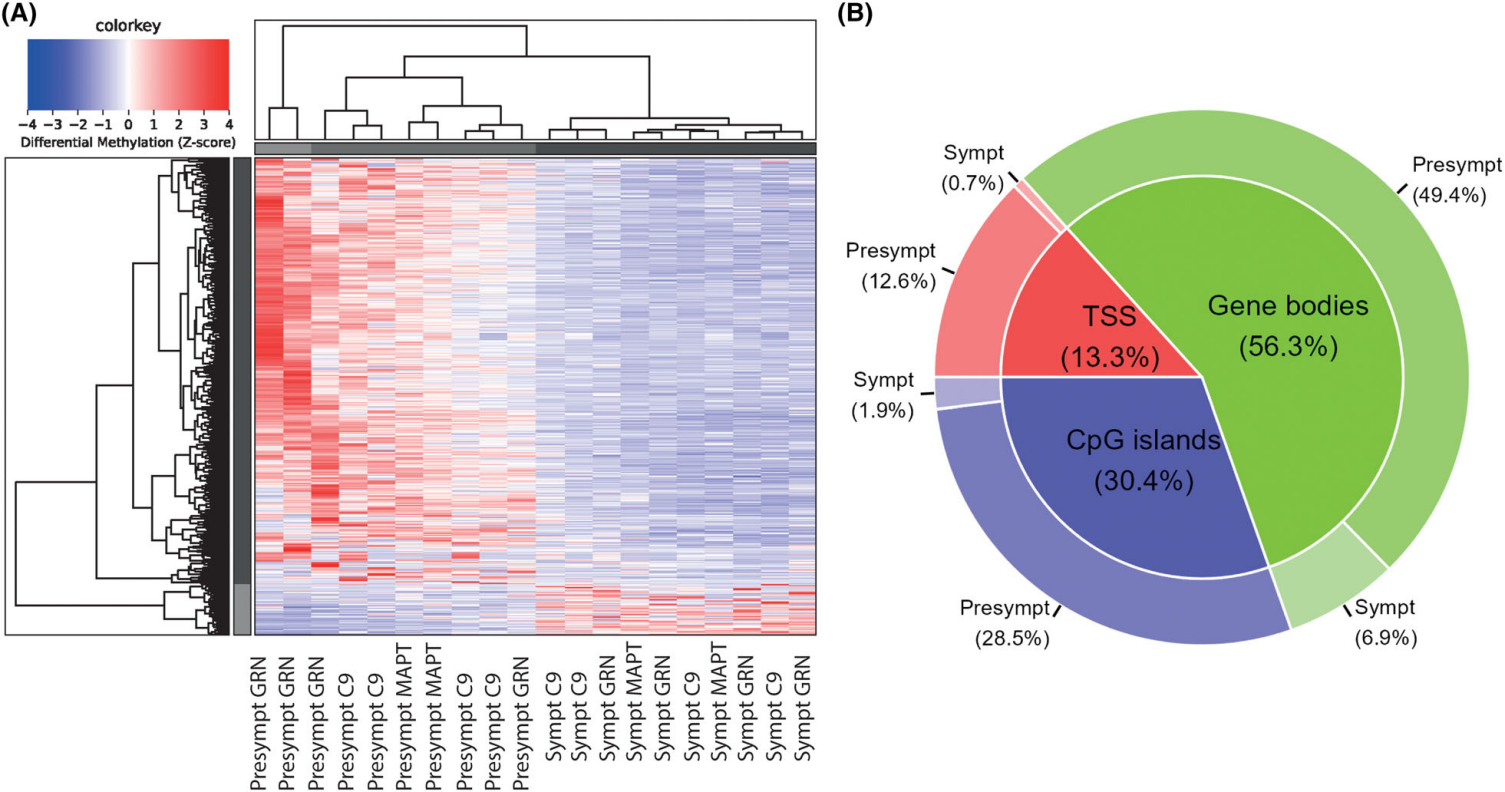
Differentially methylated regions in presymptomatic vs. symptomatic carriers. In the comparison of presymptomatic versus symptomatic carriers, 1392 significant differentially methylated regions (DMR) with a fold change ≥2 were identified, of which 1244 hypermethylated in presymptomatic carriers and 148 in symptomatic carriers (A). These DMRs included TSS (13.3%), gene bodies (56.3%), and CpG islands (30.4%) (B). Clustering shown in figure was performed based on a differential methylation z‐score, including DMRs with a fold change ≥2, excluding DMRs located on the X and Y chromosomes. Legend: C9, *C9orf72* gene; Presympt, presymptomatic; Sympt, symptomatic.

**Figure 3:**
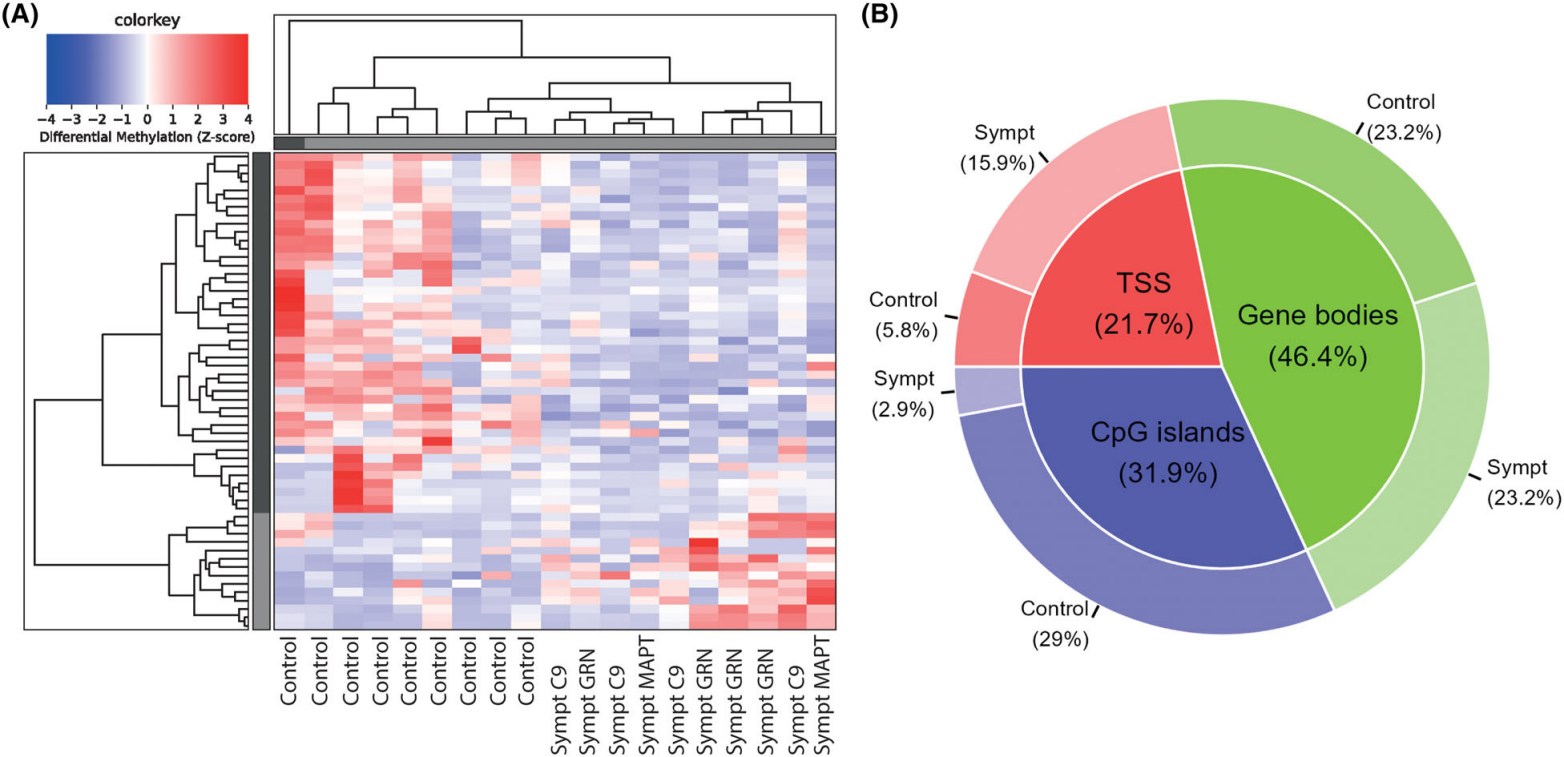
Differentially methylated regions in symptomatic carriers versus healthy controls. In the comparison of presymptomatic versus healthy controls, 57 significant differentially methylated regions (DMR) with a fold change ≥2 were identified, of which 14 hypermethylated in symptomatic carriers and 43 in healthy controls (A). These DMRs included TSS (21.7%), gene bodies (46.4%), and CpG islands (31.9%) (B). Clustering shown in figure was performed based on a differential methylation z‐score, including DMRs with a fold change ≥2, excluding DMRs located on the X and Y chromosomes. C9, *C9orf72* gene; Sympt, symptomatic.

In the derivation cohort, the comparison of presymptomatic carriers to healthy controls resulted in 838 autosomal DMRs with a fold change ≥2, including TSS (19.1%), gene bodies (52.1%), or CpG islands (28.8%), of which 678 hypermethylated in presymptomatic carriers and 160 in healthy controls (Fig. 1). The comparison of presymptomatic to symptomatic carriers resulted in 1392 autosomal DMRs with a fold change ≥2, including TSS (13.3%), gene bodies (56.3%), or CpG islands (30.4%), of which 1244 hypermethylated in presymptomatic carriers and 148 in symptomatic carriers (Fig. 2). The comparison of symptomatic carriers to healthy controls resulted in only 57 autosomal DMRs with a fold change ≥2, including TSS (21.7%), gene bodies (46.4%) or CpG islands (31.9%), of which 14 hypermethylated in symptomatic carriers and 43 in healthy controls (Fig. 3). Presymptomatic carriers therefore showed the most distinctive methylation profile compared to the other groups, characterized predominantly by hypermethylation, but also hypomethylation at specific sites. All identified DMRs and their overlap with TSS, gene bodies and CpG islands can be found in Tables S3, S4 and S5 (respectively presymptomatic vs. healthy controls, presymptomatic vs. symptomatic, and symptomatic vs. healthy controls).

### Cumulative DMR scores in derivation and validation cohorts

**Figure 4:**
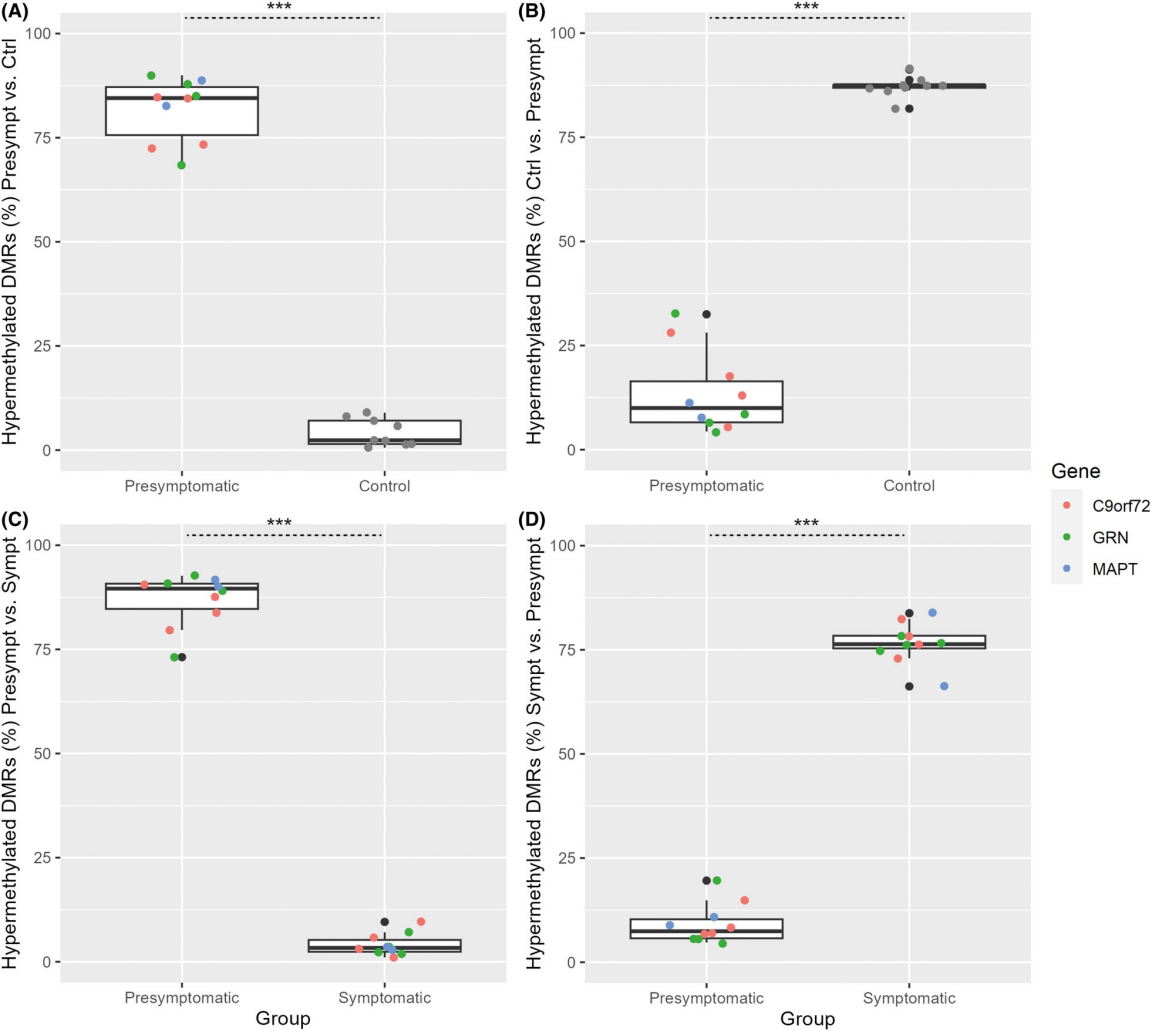
Comparisons of cumulative scores of differentially methylated regions in the derivation cohort. Cumulative scores, estimated based on DMRs in the derivation cohort, were internally validated in presymptomatic carriers versus healthy controls (A and B) and in presymptomatic carriers versus symptomatic carriers (C and D). Cumulative scores for hypermethylated DMRs of the presymptomatic group were significantly higher in presymptomatic carriers than in healthy controls (A) and symptomatic carriers (C). Conversely, cumulative scores for hypermethylated DMRs of the healthy control group (B) and of the symptomatic group (D) were significantly higher in these groups than in presymptomatic carriers. Cumulative DMR‐scores are expressed as a percentage of total scores. Data points are color coded by variant‐carrying gene in the presymptomatic and symptomatic groups. ***p < 0.001 with Mann–Whitney *U*‐test. Ctrl, healthy control group; DMR, differentially methylated region; Presympt, presymptomatic group; Sympt, symptomatic group.

In the derivation cohort, as expected, cumulative scores for hypermethylated DMRs of the presymptomatic group were significantly higher in presymptomatic carriers than in healthy controls (*p* < 0.001; Fig. 4A) and symptomatic carriers (*p* < 0.001; Fig. 4C). On the other hand, cumulative scores for hypermethylated DMRs of the healthy control group (*p* < 0.001; Fig. 4B), and cumulative scores for hypermethylated DMRs of the symptomatic group (*p* < 0.001; Fig. 4D) were significantly higher in these groups than in presymptomatic carriers.

**Figure 5:**
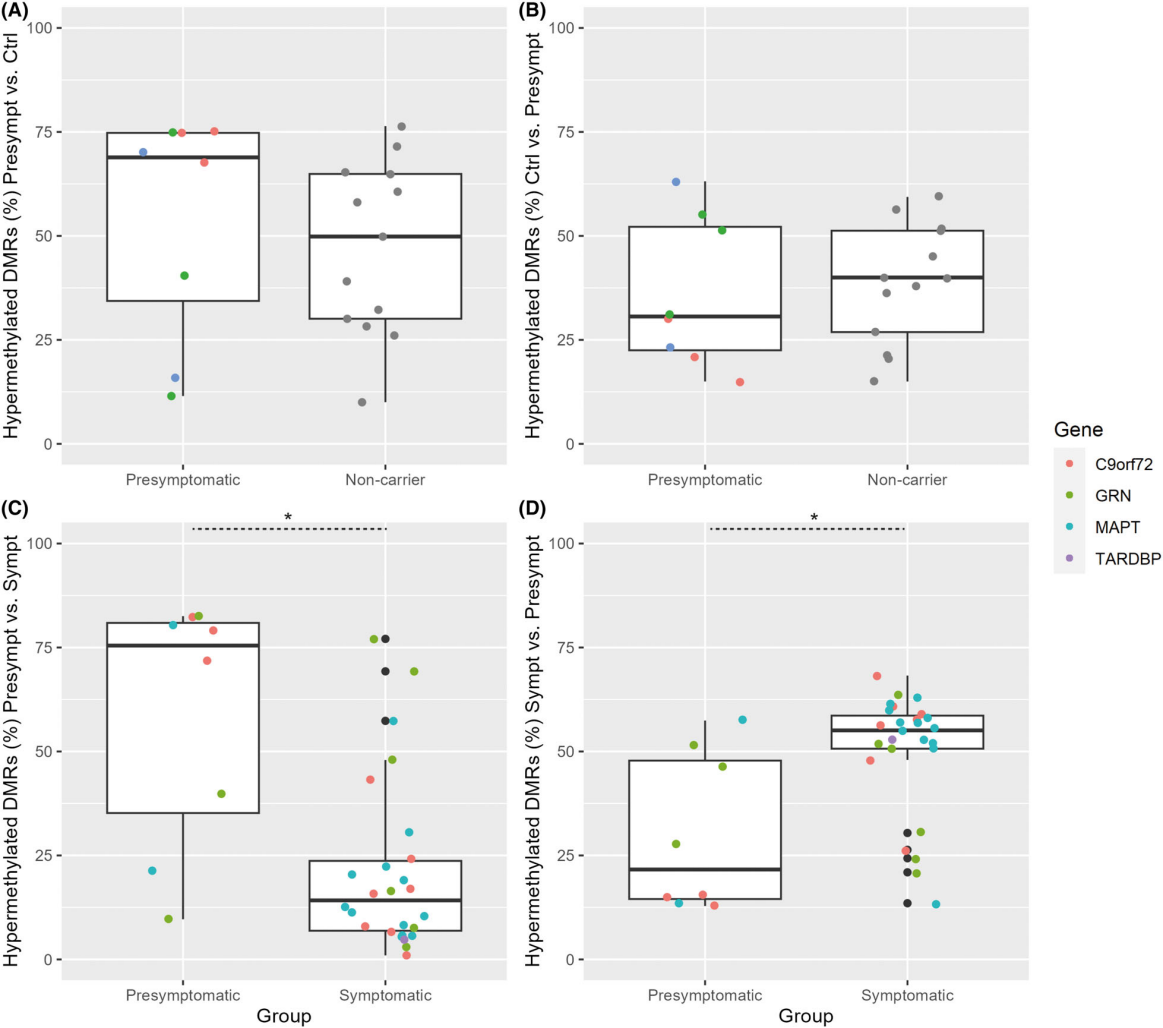
Comparisons of cumulative scores of differentially methylated regions in the validation cohort. Cumulative scores, estimated based on DMRs in the derivation cohort, were retested in an independent validation cohort of 8 presymptomatic carriers, 26 symptomatic carriers, and 13 noncarriers from genetic FTD families. Cumulative scores for hypermethylated DMRs of presymptomatic carriers and healthy controls did not significantly differ between presymptomatic carriers and noncarriers of the validation cohort (A and B). Cumulative scores for hypermethylated DMRs of the presymptomatic group (vs. symptomatic) were significantly higher in presymptomatic than in symptomatic carriers of the validation cohort (C). Cumulative scores for hypermethylated DMRs of the symptomatic group (vs. presymptomatic) were significantly higher in symptomatic than in presymptomatic carriers of the validation cohort (D). Cumulative DMR‐scores are expressed as a percentage of total scores. Data points of the presymptomatic and symptomatic groups are color‐coded by variant‐carrying gene. *p < 0.01 with Mann Whitney *U*‐test. Ctrl, healthy control group; DMR, differentially methylated region; Presympt, presymptomatic group; Sympt, symptomatic group.

Next, we reevaluated the identified DMRs in the validation cohort. Cumulative scores for hypermethylated DMRs of the presymptomatic group (vs. healthy controls) were relatively high in presymptomatic carriers of the validation cohort, but did not differ significantly from DMR scores in noncarriers (*p* = 0.328; Fig. 5A). Similarly, cumulative scores for hypermethylated DMRs of the healthy control group (vs. presymptomatic) did not significantly differ between noncarriers and presymptomatic carriers of the validation cohort (*p* = 0.717; Fig. 5B). In contrast, cumulative scores for hypermethylated DMRs of the presymptomatic group (vs. symptomatic) were significantly higher in presymptomatic than in symptomatic carriers of the validation cohort (*p* = 0.003; Fig. 5C). Consistently, cumulative scores for hypermethylated DMRs of the symptomatic group (vs. presymptomatic) were higher in symptomatic than in presymptomatic carriers of the validation cohort (*p* = 0.007; Fig. 5D).

### Protein–protein interaction analysis

PPI analysis was performed separately on methylated TSS, generally associated with reduced gene expression, and on methylated gene bodies, generally associated with increased gene expression.^50^, ^51^, ^52^ Presymptomatic carriers had TSS hypermethylation of genes involved in protein deubiquitination and ubiquitin‐dependent catabolic processes compared to both healthy controls (102 nodes, enrichment ratio = 2.6, *p* < 0.001 with false discovery rate (FDR); Table S6, Fig. S1) and symptomatic carriers (52 nodes, enrichment ratio = 54.0, FDR *p* < 0.001; Table S7, Fig. S2). In particular, genes of the ubiquitin‐specific peptidase 17 (USP17) family were implicated in both comparisons, displaying consistently hypermethylated TSS regions in the presymptomatic group (Figs. S3 and S4). Presymptomatic carriers also showed gene body hypermethylation of genes involved in cell regulation and development, including more specific terms for neurogenesis, neuron differentiation and development, compared to symptomatic carriers (594 nodes, enrichment ratio = 1.3; FDR *p*‐value <0.01; Table S8, Fig. S5), but no significant enrichment compared to healthy controls. Neither TSS nor gene body hypermethylation in healthy controls and symptomatic carriers (vs. presymptomatic carriers) showed significant biological associations.

## Discussion

In this first study of cfDNA methylation in genetic FTD, we provide preliminary evidence of differential cfDNA methylation between clinical stages of genetic FTD. Through the high‐throughput MeD‐seq technique for genome‐wide DNA methylation analysis, we identified a distinctive methylation profile of cfDNA in presymptomatic carriers of genetic FTD pathogenic variants, distinguishing them from healthy controls and symptomatic carriers. TSS hypermethylation in the presymptomatic group, generally associated with gene downregulation, was enriched for ubiquitin‐dependent processes, while gene body hypermethylation, generally associated with gene upregulation, was enriched for neuronal cell processes. These differential methylation patterns suggest the occurrence of neuronal cell death at the presymptomatic stage, as the likely source of cfDNA shed into the circulation, which however requires further validation in future studies.

The distinctive methylation profile of presymptomatic carriers, as opposed to healthy controls and symptomatic carriers, indicates that cfDNA methylation is dependent on clinical stage in genetic FTD. A prior study found stage‐dependent methylation of blood DNA in (unspecified) dementia; the presymptomatic stage (prior to dementia diagnosis) showed mostly hypermethylation compared to controls, while the symptomatic stage (after diagnosis) showed mostly hypomethylation.^29^ These changes support the hypothesis that blood DNA, or specifically cfDNA, may vary throughout the disease following the rate of cell death and other pathological processes. Importantly, by examining a heterogeneous group of different genetic subtypes of FTD, we have identified methylation changes common to all subtypes, yet clustering of presymptomatic carriers with the same variant‐carrying gene (Figs. 1 and 2) suggests subtype‐specific methylation changes. While our findings of differential cfDNA methylation in presymptomatic vs. symptomatic carriers were consistent across derivation and validation cohorts, we could not replicate the difference between presymptomatic carriers and healthy controls in the group of noncarriers from genetic FTD families. This finding raises the question as to whether shared methylation patterns could be present across members of genetic FTD families, acquired in a shared environment^53^ or passed on from earlier generations of individuals who developed the disease, as recent evidence suggests the possibility of transgenerational inheritance of methylation patterns in mammals.^54^ However, this is still highly controversial.^55^

The genomic location of methylation changes can provide clues on the tissue origin of cfDNA. The establishment of tissue‐specific DNA methylation occurs during embryogenesis, such that methylation especially of CpG island‐associated gene TSS leads to the repression of gene transcription.^51^, ^56^ In contrast, intragenic DNA methylation changes occur mainly as a consequence of transcriptional changes, and gene body hypermethylation is associated with increased transcription.^52^, ^57^ In our study, the finding of differential methylation of many CpG islands and TSS in presymptomatic carriers is therefore highly suggestive of the presence of a different (non‐hematological) cell type contributing to blood cfDNA, rather than the occurrence of transcriptional changes in blood cells only.

We identified TSS hypermethylation (suggesting gene downregulation) of genes from the family of USP17, a deubiquitinating enzyme that has been implicated in several cellular functions, such as cell cycle progression,^58^ chemokine‐driven cell motility, endocytosis, and peripheral lysosome trafficking.^59^ This enzyme has not been previously associated with FTD; however, ubiquitin‐dependent signaling is a well‐known disease‐related process in several neurodegenerative disorders, contributing to protein degradation, regulation of neuronal function, and inflammation.^60^ A recent study of brain methylation in symptomatic postmortem FTLD has also identified differential methylation of a deubiquinating enzyme (*OTUD4*), postulating a role for methylation in key disease processes such as ubiquitin signaling.^38^ Therefore, TSS hypermethylation in the presymptomatic group may indicate the occurrence of disease‐associated mechanisms at this stage. At the same time, presymptomatic carriers showed gene body methylation (suggesting gene upregulation) of genes involved in neuronal cellular processes when compared to symptomatic carriers, including the *TARDBP* and the *PRKAR1B* genes, both associated with FTD.^61^, ^62^ Gene body methylation may also show tissue‐specific profiles, likely related to transcriptional changes.^63^, ^64^, ^65^, ^66^, ^67^ Interestingly, three of the genes displaying gene body hypermethylation (*CACNA1A*, *PCDH9*, and *SHANK3*) in our group of presymptomatic carriers (vs. symptomatic carriers) have been previously associated with neuronal tissue‐specific methylation,^67^ suggesting that at least part of the presymptomatic methylation profile has a neuronal origin. The presence of a neuronal methylation profile is consistent with our *a priori* hypotheses, and may be indicative of increased neuronal cell death in the presymptomatic stage. Upregulated processes (neurodevelopment and neurogenesis) furthermore suggest the possible occurrence of neuronal compensatory mechanisms preceding the onset of disease, similar to changes described in the early stages of AD.^50^ The finding that symptomatic carriers do not differ greatly from healthy controls is unexpected and requires further attention. It is possible that symptomatic carriers lose the methylation profile characteristic of presymptomatic carriers, as neurons decrease in quantity or become increasingly dysfunctional due to more advanced neurodegeneration. Alternatively, it may be that different implicated cell types (neurons, glia, etc.) contribute to cfDNA release, hampering the detection of a clear cellular signature.

Besides these associations, the origin of cfDNA and the underlying biology of differential methylation in FTD is still in part unknown, which may be due to several reasons. First, in contrast to cancer cfDNA, where one population of cancer cells can be identified through specific cancer‐associated mutations,^13^ it is possible that cfDNA in neurodegeneration originates from different implicated cells, such as glial cells next to neurons. Indeed, a prior study in AD brains found the greatest number of differentially methylated loci in microglia and astrocytes, rather than neurons^28^; this could lead to a mixed methylation profile in blood cfDNA. Second, methylation has been shown to vary regionally in the brain^27^ and according to neuropathological disease stage in AD,^26^ which could make the methylation profile less distinguishable especially in symptomatic carriers with widespread neurodegeneration. Third, variability in cfDNA profile may stem from the partly different pathophysiologies of genetic and pathological subtypes of FTD. Based on the clustering of carriers of specific genes (Figs. 1 and 2), it appears highly likely that methylation patterns differ to some extent between genetic groups, or between groups with the same pathology (*MAPT* vs. *C9orf72/GRN*). However, this hypothesis still needs to be properly tested in larger cohorts with sufficient sample sizes for each subtype. For the above reasons, it is too premature to speak of biomarker applications for cfDNA in FTD, but future studies may uncover its potential in targeted studies in specific stages and subtypes of FTD, through the comparison of methylated loci to emerging libraries of cell‐specific methylation markers^68^ or through the application of MeD‐seq analysis to single nuclei. Recent single‐nucleus transcriptomic studies in brain tissue from FTD subtypes have been extremely insightful,^69^, ^70^ and could be paired with methylation data. Due to the rarity of autopsies in the presymptomatic stage of FTD, however, it may be difficult to validate presymptomatic findings in brain tissue.

Our novel study of cfDNA in FTD relied upon the use of a high‐throughput technique for genome‐wide methylation profiling, MeD‐seq,^43^ which enabled to make correlations between high numbers of methylated sites and biological networks. However, some limitations should be considered. Pre‐analytical variability may arise from our methods of cfDNA collection, including two slightly different protocols using either EDTA or CellSave tubes. Due to this methodological aspect, we could not examine the absolute levels of cfDNA in plasma, which may vary using different collection tubes. Both methods were, however, found to yield similar methylation outcomes using MeD‐seq, granted that a sufficient amount of cfDNA is isolated.^48^ The lower age at sample collection of some healthy controls in the derivation cohort may provide age‐related bias. However, by focusing on consistent DNA methylation differences between all individual samples in the groups and by including healthy controls from a wide range of ages, we excluded the identification of regions associated with age only. Further, cumulative DMR scores did not correlate with age neither in the derivation nor in the validation cohort, arguing against the presence of an important age‐related effect. While our findings suggest that methylation is stage‐dependent in FTD, our current observations are merely cross‐sectional and based on a mixed group of genetic FTD. In future studies, longitudinal data could provide additional insights into how the methylation profile develops throughout the disease course, and methylation profiles should be compared between participants with different subtypes of FTD to identify gene‐ or pathology‐associated patterns. To understand the biological and clinical relevance of cfDNA methylation in FTD, future studies should also investigate the relationship between DMR scores and markers of neuronal dysfunction (e.g., FDG‐PET and arterial spin labeling MRI) and neurodegeneration (e.g., structural MRI and plasma/CSF neurofilament light chain).

To conclude, a distinctive methylation profile of plasma‐derived cfDNA methylation is associated with the presymptomatic stage of FTD. This preliminary evidence in genetic FTD should be validated in larger cohorts of genetic and sporadic FTD, and preferentially within genetic and pathological subtypes that may have heterogeneous biological underpinnings of cfDNA release into the circulation. While the clinical application of cfDNA methylation as a biomarker in FTD is still a few steps away, its potential to uncover disease mechanisms and biological targets motivates further investigation and validation of this marker.

## Author Contributions

Lucia A. A. Giannini, Ruben G. Boers, JG and John C. van Swieten contributed to the conception of the study design. Lucia A. A. Giannini, Ruben G. Boers, Emma L. van der Ende, Joachim B. Boers, Wilfred F. J. van IJcken, Lieke H. Meeter, Jeroen G. J. van Rooij, Wiep Scheper, Joost Gribnau and John C.van Swieten contributed to data collection, analysis and interpretation. Lucia A. A. Giannini, Emma L. van der Ende, Jackie M. Poos, Lize C. Jiskoot, Elise G. Dopper, Yolande A. L. Pijnenburg, Harro Seelaar and John C. van Swieten provided substantial contributions to patient recruitment and clinical characterization. Lucia A. A. Giannini and Ruben G.Boers drafted the manuscript. All authors critically revised the manuscript for content.

## Conflicts of Interest

The authors declare no conflict of interest or financial interests except for RGB, JBB, WFJvIJ, and JG, who report being shareholder in Methylomics B.V., a commercial company that applies MeD‐seq to develop methylation markers for cancer staging.

## Supporting information


Table S1.

Figure S1.
