# Supplementary material for: Distinctive cell‐free DNA methylation characterizes presymptomatic genetic frontotemporal dementia
Source: Ann Clin Transl Neurol. 2024 Mar 13;11(3):744–56. doi: 10.1002/acn3.51997 (PMC10963298; doi:10.1002/acn3.51997)

**Online supplement for:**

**Distinctive cell-free DNA methylation characterizes presymptomatic genetic frontotemporal dementia**

Lucia AA Giannini^1^, MD, Ruben G Boers^2^, MD, Emma L van der Ende^1,3^, MD, PhD, Jackie M Poos^1^, PhD, Lize C Jiskoot^1^, PhD, Joachim B Boers^2^, MSc, Wilfred FJ van Ijcken^4^, PhD, Elise G Dopper^1^, MD, PhD, Yolande AL Pijnenburg^5,6^, MD, PhD, Harro Seelaar^1^, MD, PhD, Lieke H Meeter^1^, MD, PhD, Jeroen GJ van Rooij^1^, PhD, Wiep Scheper^6,7,8^, PhD, Joost Gribnau^2^, PhD, John C van Swieten^1^, MD, PhD

^1^Department of Neurology, Alzheimer Center Erasmus MC, Erasmus University Medical Center, Rotterdam, The Netherlands

^2^Department of Developmental Biology, Erasmus Medical Center, Rotterdam, The Netherlands

^3^Department of Clinical Chemistry, Amsterdam Neuroscience, Amsterdam UMC, Vrije Universiteit, Amsterdam, The Netherlands

^4^Erasmus Center for Biomics, Erasmus University Medical Center, Rotterdam, The Netherlands

^5^Alzheimer Center Amsterdam, Neurology, Vrije Universiteit, Amsterdam UMC location Vumc, Amsterdam, The Netherlands

^6^Amsterdam Neuroscience, Neurodegeneration, Amsterdam, The Netherlands

^7^Department of Functional Genomics, Center for Neurogenomics and Cognitive Research, Faculty of Science, Vrije Universiteit, Amsterdam, The Netherlands

^8^Department of Human Genetics, Vrije Universiteit, Amsterdam UMC location Vumc, Amsterdam, The Netherlands

Please send correspondence to:

*John C. van Swieten, MD, PhD

Department of Neurology, Alzheimer Center Erasmus MC

Erasmus University Medical Center

Doctor Molewaterplein 40

3015 GD Rotterdam

[j.c.vanswieten@erasmusmc.nl](mailto:j.c.vanswieten@erasmusmc.nl)

**SUPPLEMENTARY TABLES’ TITLES AND LEGENDS**

**Supplementary Table 1. Overview of demographic information and family relations in the derivation cohort**

Legend: bvFTD = behavioral variant frontotemporal dementia; FTD-ALS = frontotemporal dementia in combination with amyotrophic lateral sclerosis; HC = healthy controls; PPA = primary progressive aphasia; Presympt = presymptomatic carriers; Sympt = symptomatic carriers. For each individual of the derivation cohort, basic demographic and clinical information, including family relations to other participants, is shown.

**Supplementary Table 2. Overview of demographic information and family relations in the validation cohort**

Legend: bvFTD = behavioral variant frontotemporal dementia; FTD-ALS = frontotemporal dementia in combination with amyotrophic lateral sclerosis; NC = non-carriers; PPA = primary progressive aphasia; Presympt = presymptomatic carriers; Sympt = symptomatic carriers. For each individual of the validation cohort, basic demographic and clinical information, including family relations to other participants, is shown.

**Supplementary Table 3. Identified differentially methylated regions in presymptomatic carriers vs. healthy controls**

Legend: DMR = differentially methylated region; postTSS1KB-TES = regions starting at 1 Kb after the transcription start site (TSS) until the transcription end site (TES) corresponding to the gene body; TSS = transcription start site. Chromosome loci, fold change and overlapping genes or CpG islands are listed for each DMR.

**Supplementary Table 4. Identified differentially methylated regions in presymptomatic carriers vs. symptomatic carriers**

Legend: DMR = differentially methylated region; postTSS1KB-TES = regions starting at 1 Kb after the transcription start site (TSS) until the transcription end site (TES) corresponding to the gene body; TSS = transcription start site. Chromosome loci, fold change and overlapping genes or CpG islands are listed for each DMR.

**Supplementary Table 5. Identified differentially methylated regions in symptomatic carriers vs. healthy controls**

Legend: DMR = differentially methylated region; postTSS1KB-TES = regions starting at 1 Kb after the transcription start site (TSS) until the transcription end site (TES) corresponding to the gene body; TSS = transcription start site. Chromosome loci, fold change and overlapping genes or CpG islands are listed for each DMR.

**Supplementary Table 6. Protein-protein interaction analysis outcomes of TSS methylation in presymptomatic carriers vs. healthy controls**

Legend: FDR = false discovery rate; GO = Gene Ontology; PPI = protein-protein interaction; TSS = transcription start site. PPI outcomes and significantly associated GO terms for biological processes, molecular functions and cellular components are shown.

**Supplementary Table 7. Protein-protein interaction analysis outcomes of TSS methylation in presymptomatic carriers vs. symptomatic carriers**

Legend: FDR = false discovery rate; GO = Gene Ontology; PPI = protein-protein interaction; TSS = transcription start site. PPI outcomes and significantly associated GO terms for biological processes, molecular functions and cellular components are shown.

**Supplementary Table 8. Protein-protein interaction analysis outcomes of gene body methylation in presymptomatic carriers vs. symptomatic carriers**

Legend: FDR = false discovery rate; GO = Gene Ontology; PPI = protein-protein interaction. PPI outcomes and significantly associated GO terms for biological processes, molecular functions and cellular components are shown.

**Supplementary Figure 1. Protein-protein interaction network graphic of TSS methylation in presymptomatic carriers vs. healthy controls**


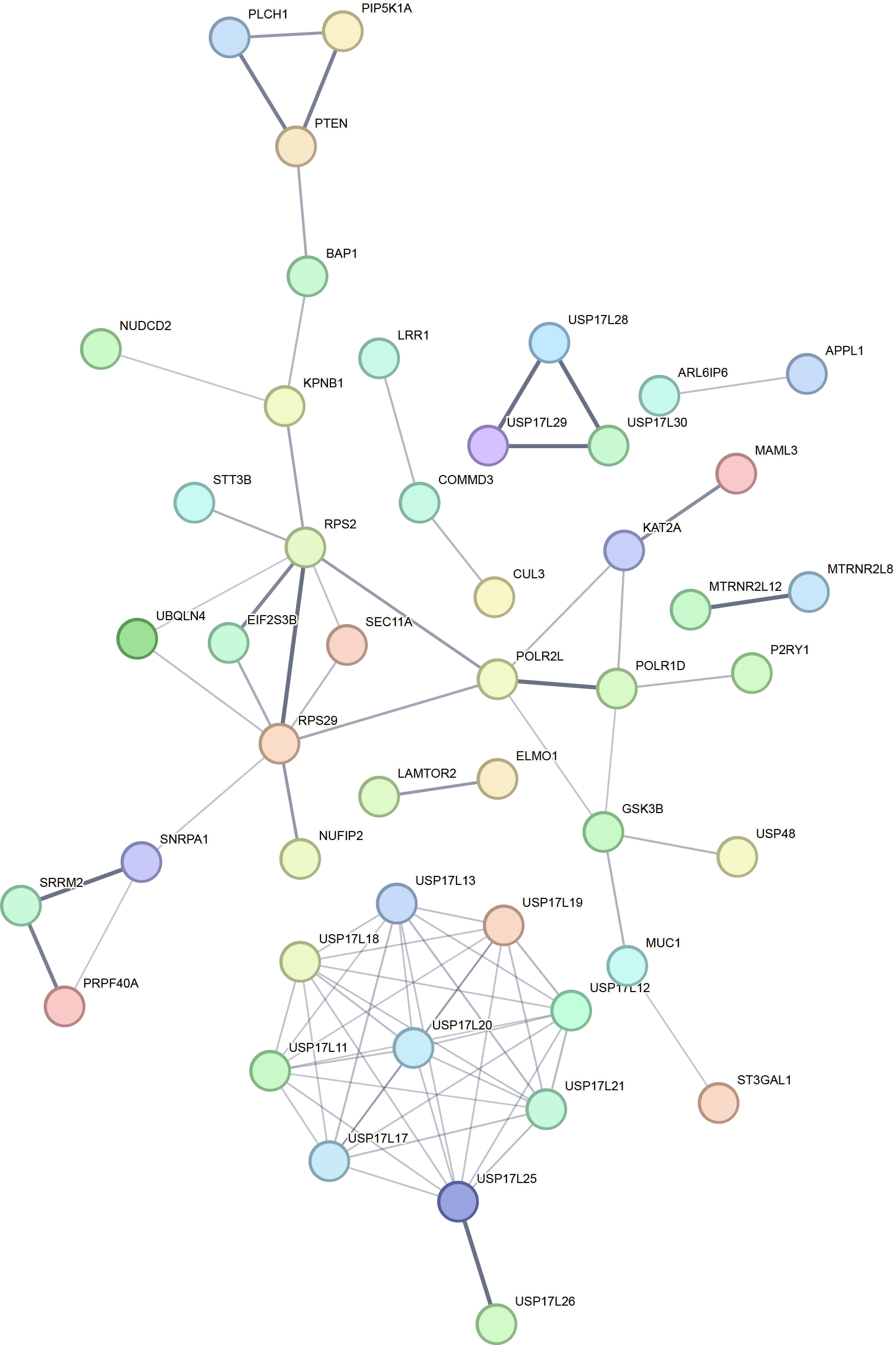


The protein-protein interaction network shown in figure has been produced using STRING (<https://string-db.org>), v. 11.5. Only connected nodes are shown. The line thickness indicates the strength of data support for the network edges.

**Supplementary Figure 2. Protein-protein interaction network graphic of TSS methylation in presymptomatic vs. symptomatic carriers**


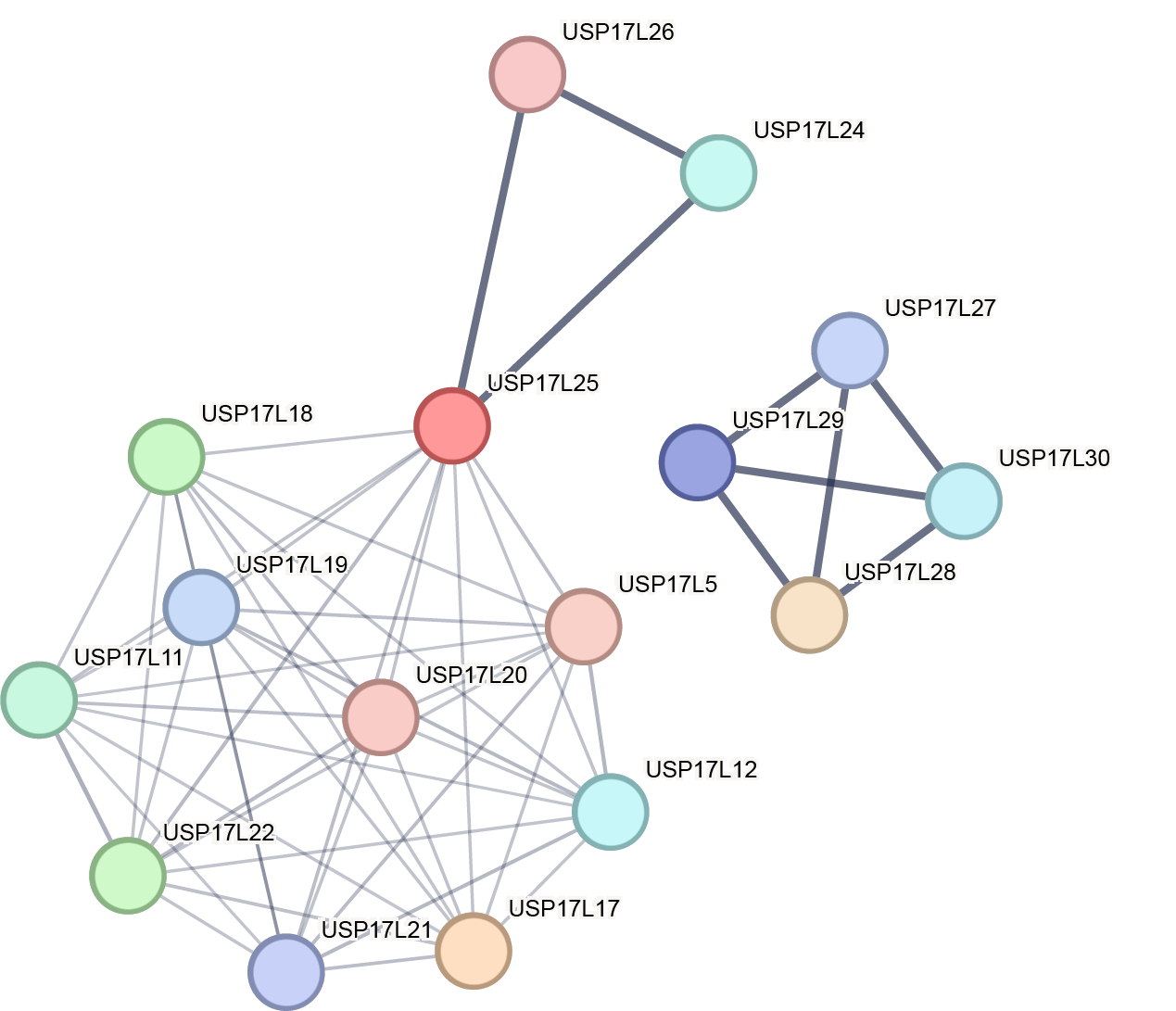


The protein-protein interaction network shown in figure has been produced using STRING (<https://string-db.org>), v. 11.5. Only connected nodes are shown. The line thickness indicates the strength of data support for the network edges.

**Supplementary Figure 3. MeD-seq reads of methylated TSS sites in genes of the USP17 family in the presymptomatic and healthy control groups**

The number of MeD-seq reads of methylated TSS sites in USP17 genes is visualized using the Integrative Genomics Viewer per individual in each row (presymptomatic carriers and healthy controls). Legend: C9 = *C9orf72* gene; Presymp = presymptomatic group.


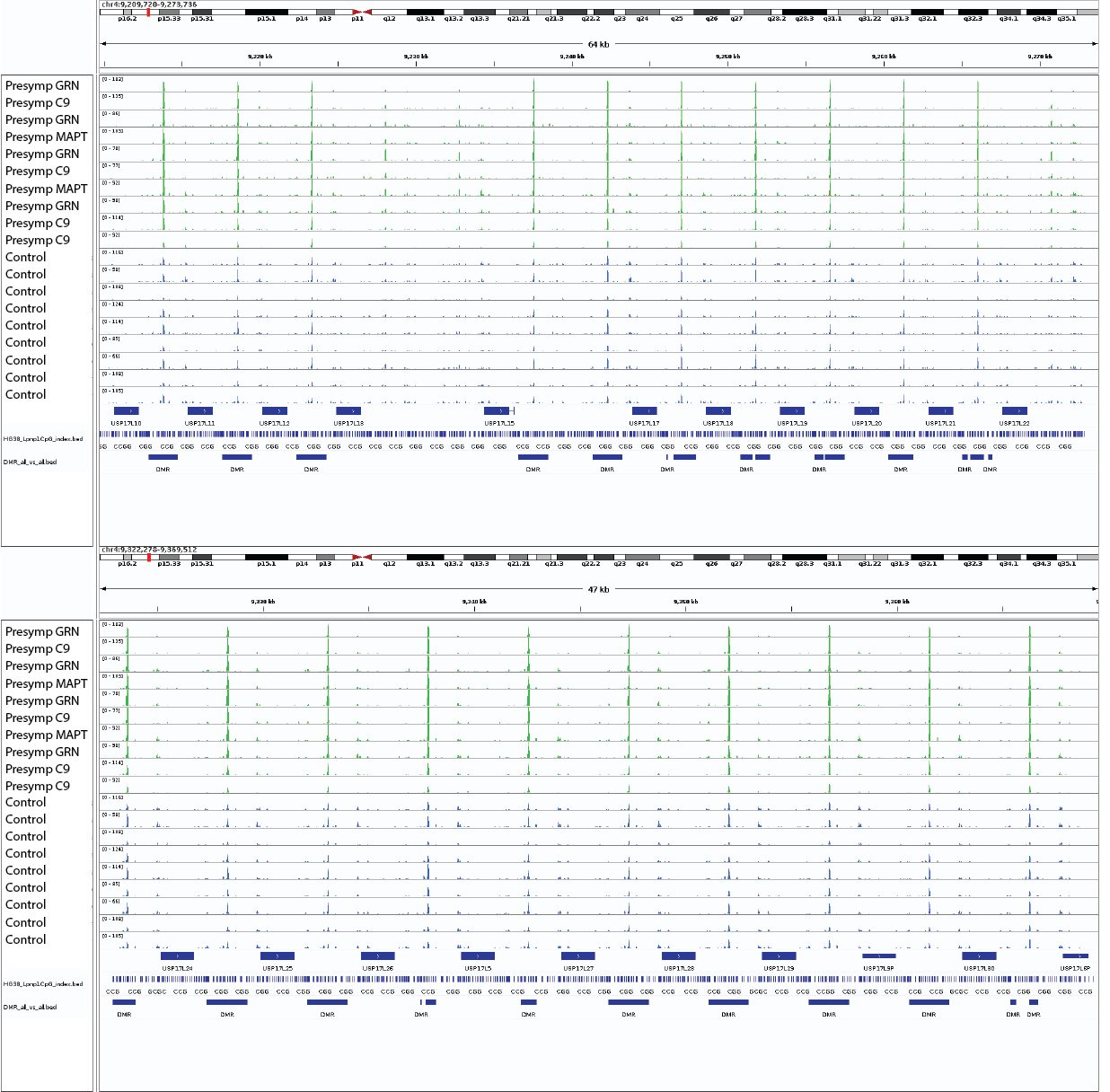


**Supplementary Figure 4. MeD-seq reads of methylated TSS sites in genes of the USP17 family in the presymptomatic and symptomatic groups**

**
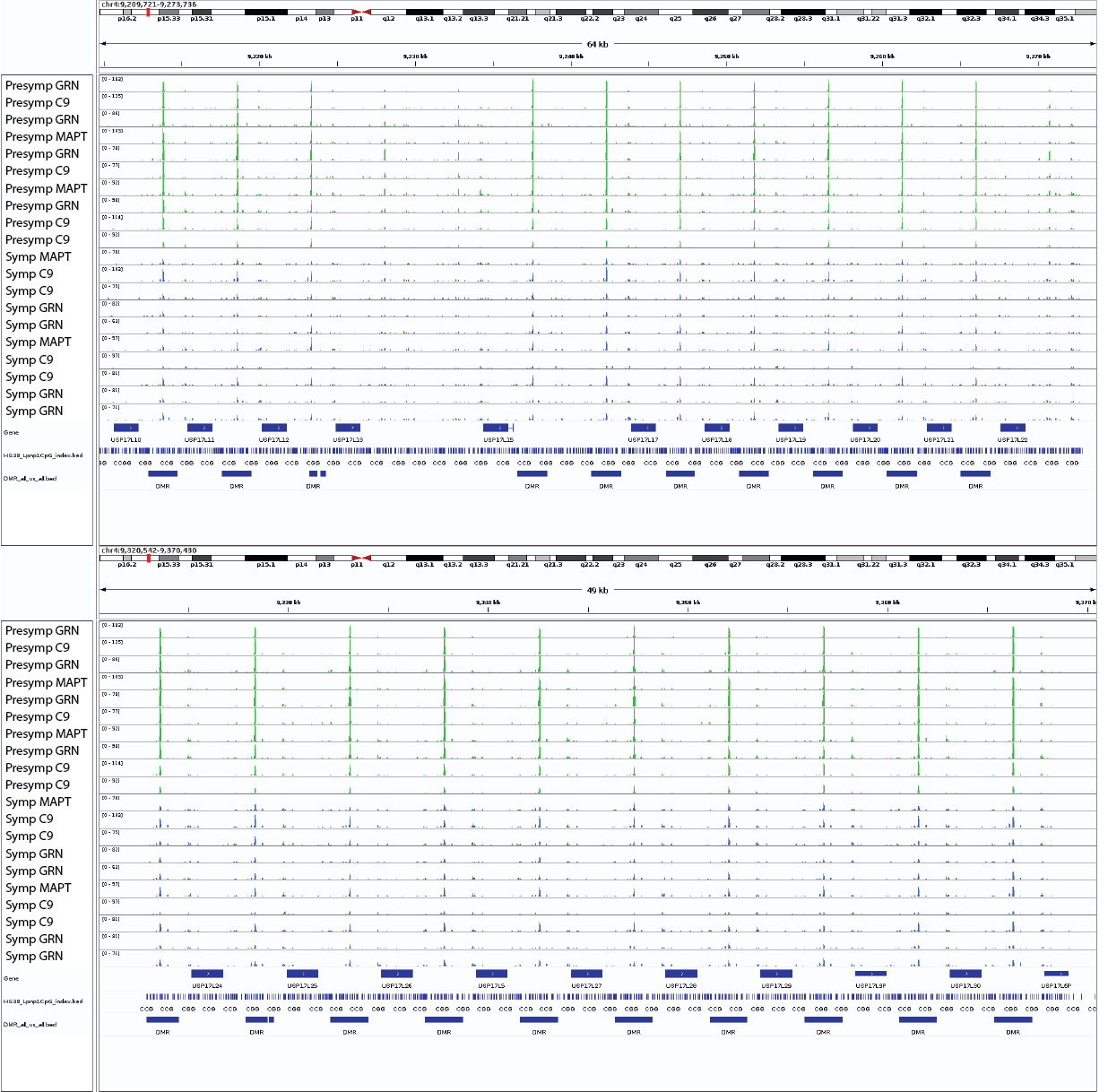
** The number of MeD-seq reads of methylated TSS sites in USP17 genes is visualized using the Integrative Genomics Viewer per individual in each row (presymptomatic and symptomatic carriers). Legend: C9 = *C9orf72* gene; Presymp = presymptomatic group; Symp = symptomatic group.

**Supplementary Figure 5. Protein-protein interaction network graphic of gene body methylation in presymptomatic vs. symptomatic carriers**

The protein-protein interaction network shown in figure has been produced using STRING (<https://string-db.org>), v. 11.5. Only connected nodes are shown. The line thickness indicates the strength of data support for the network edges.


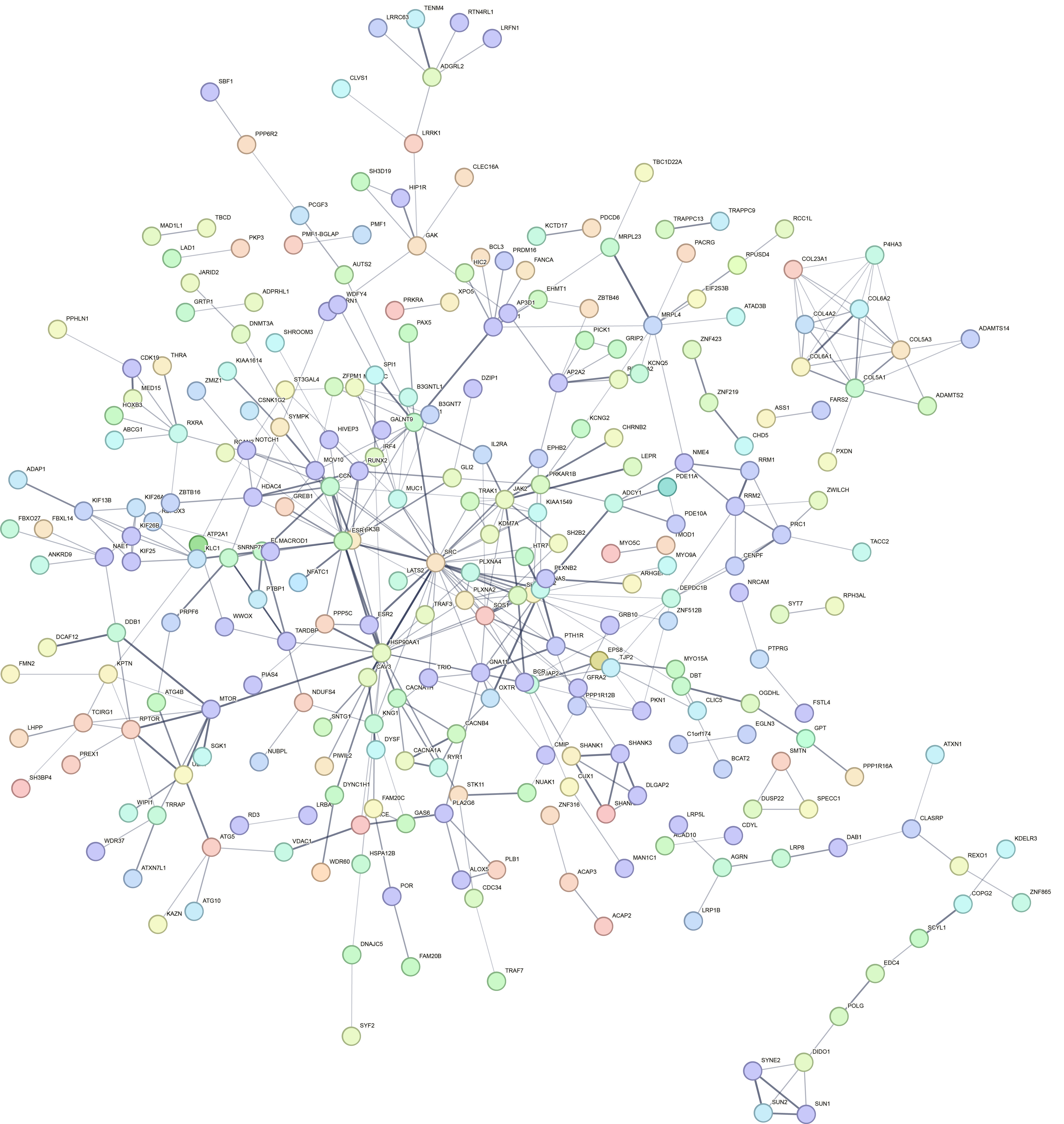

Supplement: Supplementary file 2 — Figure S1. [file ACN3-11-744-s002.docx]
